# {4-[(7-Chloro-4-quinol­yl)amino]-*N*,*N*-di­ethyl­penta­naminium}(triphenyl­phos­phine)gold(I) dinitrate

**DOI:** 10.1107/S1600536810031144

**Published:** 2010-08-18

**Authors:** Marina Hitosugi-Levesque, Joseph M. Tanski

**Affiliations:** a Department of Chemistry, Vassar College, Poughkeepsie, NY 12604, USA

## Abstract

The title compound, [Au(C_18_H_27_ClN_3_)(C_18_H_15_P)](NO_3_)_2_, is a coordination complex of gold(I) triphenyl­phosphine with the N atom in the quinoline ring of the common anti­malarial compound chloro­quine (CQ). The pendant diethyl­amino group of the CQ ligand was found to be protonated. The complex exhibits a nearly linear coordination geometry around the Au^I^ atom [N—Au—P = 176.94 (6)°], with Au—N and Au—P bond lengths of 2.070 (2) and 2.2338 (7) Å, respectively. The diethylammonium group and one of the two nitrate counter-ions are disordered with occupancy ratios of 0.519 (4):0.481 (4). The nitrate anions are hydrogen bound to both the amino and ammonium groups of the *N*,*N*-diethylpentanaminium fragment of the CQ.

## Related literature

For related structures, see: Karle & Karle (1988 ▶); Oleksyn & Serda (1993 ▶); Orlow *et al.* (2005 ▶); Borissova *et al.* (2008 ▶); Thwaite *et al.* (2004 ▶). For background to the metal coordination chemistry of chloro­quine, see: Sánchez-Delgado *et al.* (1996 ▶); Navarro *et al.* (1997 ▶, 2004 ▶). Widespread use of CQ has led to cross-resistance, limiting the efficacy of CQ-related treatments for malaria, see: World Health Organization (2010 ▶).
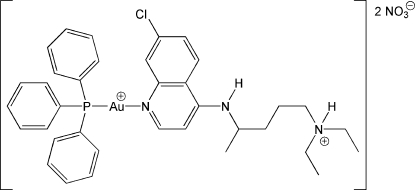


## Experimental

### 

#### Crystal data

[Au(C_18_H_27_ClN_3_)(C_18_H_15_P)](NO_3_)_2_
*M* *_r_* = 904.13
Triclinic, 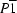

*a* = 10.2456 (5) Å
*b* = 13.1914 (6) Å
*c* = 13.6301 (7) Å
α = 85.866 (1)°
β = 88.126 (1)°
γ = 86.510 (1)°
*V* = 1833.22 (15) Å^3^
*Z* = 2
Mo *K*α radiation
μ = 4.18 mm^−1^
*T* = 125 K
0.34 × 0.14 × 0.05 mm

#### Data collection

Bruker APEXII CCD area-detector diffractometer
Absorption correction: multi-scan (*SADABS*; Bruker, 2007 ▶) *T*_min_ = 0.331, *T*_max_ = 0.818
25641 measured reflections
10116 independent reflections
8781 reflections with *I* > 2σ(*I*)
*R*_int_ = 0.030

#### Refinement

*R*[*F* ^2^ > 2σ(*F* ^2^)] = 0.027
*wR*(*F* ^2^) = 0.059
*S* = 1.02
10116 reflections
535 parameters
1 restraint
H atoms treated by a mixture of independent and constrained refinement
Δρ_max_ = 0.79 e Å^−3^
Δρ_min_ = −0.84 e Å^−3^

### 

Data collection: *APEX2* (Bruker, 2007 ▶); cell refinement: *SAINT* (Bruker, 2007 ▶); data reduction: *SAINT*; program(s) used to solve structure: *SHELXS97* (Sheldrick, 2008 ▶); program(s) used to refine structure: *SHELXL97* (Sheldrick, 2008 ▶); molecular graphics: *SHELXTL* (Sheldrick, 2008 ▶); software used to prepare material for publication: *SHELXTL*.

## Supplementary Material

Crystal structure: contains datablocks I, global. DOI: 10.1107/S1600536810031144/pv2313sup1.cif

Structure factors: contains datablocks I. DOI: 10.1107/S1600536810031144/pv2313Isup2.hkl

Additional supplementary materials:  crystallographic information; 3D view; checkCIF report

## Figures and Tables

**Table 1:** Hydrogen-bond geometry (Å, °)

*D*—H⋯*A*	*D*—H	H⋯*A*	*D*⋯*A*	*D*—H⋯*A*
N5—H5⋯O4	0.93	1.77	2.70 (2)	177
N5′—H5′⋯O4	0.93	2.00	2.81 (2)	144
N4—H4⋯O2′	0.84 (2)	2.28 (2)	3.030 (6)	149 (3)
N4—H4⋯O3^i^	0.84 (2)	2.32 (3)	3.013 (5)	140 (3)

Symmetry code: (i) 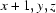.

**Fig. 1.**
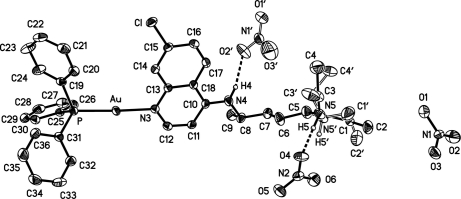
A view of the title complex, with displacement ellipsoids shown at the 50% probability level. H atoms on carbon have been omitted for clarity; primed atoms represent the disordered fraction of the NO3 ion and the diethylammonium group.

## supplementary crystallographic information

### Comment

Chloroquine (CQ) has been used in treatments against the malaria parasites *Plasmodium falciparum* and *Plasmodium vivax*. Wide use of CQ has led to cross-resistance, limiting the efficacy of CQ related treatments. (World Health Organization, 2010). Complexing CQ with metal containing fragments such as gold(I) triphenylphosphine and gold (III) tetrachloride can enhance its ability to combat CQ-resistant strains of malaria (Navarro *et al.* 1997; Navarro *et al.* 2004), as can CQ complexes of Rh and Ru (Sánchez-Delgado *et al.* 1996). While some of the biological effects of coordinating metals to chloroquine and ts derivatives have been reported, a lack of structure-activity correlation remains.

In the title complex (Fig. 1) gold(I) is coordinated to the nitrogen atom in the quinoline ring as proposed earlier (Navarro *et al.* 1997). The complex exhibits a nearly linear coordination geometry around gold, with an N—Au—P angle of 176.94 (6)°. The Au—N and Au—P bond distance are 2.070 (2) Å and 2.2338 (7) Å, respectively. These bond distances are similar to those found in thre structure of (triphenylphosphine)gold(I) chloride, with Au—P 2.2313 (4) Å (Borissova *et al.*, 2008), as well as the Au—N and Au—P distances in the structure of the cationic pyridine adduct (triphenylphosphine)gold(I) pyridine tetrafluoroborate, with Au—N 2.073 (3) Å and Au—P 2.2364 (8) Å (Thwaite *et al.*, 2004). The pendant diethylamino group (N4) of the CQ ligand in the title complex was found to be protonated, and the nitrate anions are hydrogen bound to both the N1 amino and N4 ammonium groups of the 1,4-pentanediamine fragment of the CQ.

### Experimental

[(CQ)Au(PPh_3_)][NO_3_] was prepared from chloroquine (CQ) free base and triphenylphosphine (PPh_3_) according to literature procedure (Navarro *et al.*, 2004). The protonated title complex, [C_36_H_42_AuClPN_3_](NO_3_)_2_ was obtained by diffusion of diethyl ether into an acetone solution of [(CQ)Au(PPh_3_)][NO_3_] over a period of two weeks. All manipulations were carried out under nitrogen using common Schlenck technique. X-ray diffraction quality crystals were separated as colorless plates.

### Refinement

Hydrogen atoms on carbon were included in calculated positions and were refined using a riding model at C–H = 0.95, 0.98 and 0.99 Å and *U*_iso_(H) = 1.2, 1.5 and 1.2 × *U*_eq_(C) of the aryl, methyl and methylene C-atoms, respectively. The hydrogen atom on N4 was refined semi-freely with the help of a distance restraint at N–H = 0.88 Å and *U*_iso_(H) = 1.2 × *U*_eq_(N). The presence of a residual electron density peak and the proximity of a nitrate anion indicated that N5 was protonated. Hydrogen atoms on N5 were included in calculated positions and were refined using a riding model at N–H = 0.93 Å and *U*_iso_(H) = 1.2 × *U*_eq_(N) .

### Crystal data

**Table:**

[Au(C_18_H_27_ClN_3_)(C_18_H_15_P)](NO_3_)_2_	*Z* = 2
*M**_r_* = 904.13	*F*(000) = 904
Triclinic, *P*1	*D*_x_ = 1.638 Mg m^−^^3^
Hall symbol: -P 1	Mo *K*α radiation, λ = 0.71073 Å
*a* = 10.2456 (5) Å	Cell parameters from 9914 reflections
*b* = 13.1914 (6) Å	θ = 2.6–28.2°
*c* = 13.6301 (7) Å	µ = 4.18 mm^−^^1^
α = 85.866 (1)°	*T* = 125 K
β = 88.126 (1)°	Plate, colourless
γ = 86.510 (1)°	0.34 × 0.14 × 0.05 mm
*V* = 1833.22 (15) Å^3^	

### Data collection

**Table:**

Bruker APEXII CCD area-detector diffractometer	10116 independent reflections
Radiation source: fine-focus sealed tube	8781 reflections with *I* > 2σ(*I*)
graphite	*R*_int_ = 0.030
φ and ω scans	θ_max_ = 30.4°, θ_min_ = 1.5°
Absorption correction: multi-scan (*SADABS*; Bruker, 2007)	*h* = −13→14
*T*_min_ = 0.331, *T*_max_ = 0.818	*k* = −18→18
25641 measured reflections	*l* = −18→19

### Refinement

**Table:**

Refinement on *F*^2^	Primary atom site location: structure-invariant direct methods
Least-squares matrix: full	Secondary atom site location: difference Fourier map
*R*[*F*^2^ > 2σ(*F*^2^)] = 0.027	Hydrogen site location: inferred from neighbouring sites
*wR*(*F*^2^) = 0.059	H atoms treated by a mixture of independent and constrained refinement
*S* = 1.02	*w* = 1/[σ^2^(*F*_o_^2^) + (0.0268*P*)^2^ + 0.224*P*] where *P* = (*F*_o_^2^ + 2*F*_c_^2^)/3
10116 reflections	(Δ/σ)_max_ = 0.002
535 parameters	Δρ_max_ = 0.79 e Å^−^^3^
1 restraint	Δρ_min_ = −0.84 e Å^−^^3^

### Special details

**Table:**

Geometry. All e.s.d.'s (except the e.s.d. in the dihedral angle between two l.s. planes) are estimated using the full covariance matrix. The cell e.s.d.'s are taken into account individually in the estimation of e.s.d.'s in distances, angles and torsion angles; correlations between e.s.d.'s in cell parameters are only used when they are defined by crystal symmetry. An approximate (isotropic) treatment of cell e.s.d.'s is used for estimating e.s.d.'s involving l.s. planes.
Refinement. Refinement of *F*^2^ against ALL reflections. The weighted *R*-factor *wR* and goodness of fit *S* are based on *F*^2^, conventional *R*-factors *R* are based on *F*, with *F* set to zero for negative *F*^2^. The threshold expression of *F*^2^ > σ(*F*^2^) is used only for calculating *R*-factors(gt) *etc*. and is not relevant to the choice of reflections for refinement. *R*-factors based on *F*^2^ are statistically about twice as large as those based on *F*, and *R*- factors based on ALL data will be even larger.

### Fractional atomic coordinates and isotropic or equivalent isotropic displacement parameters (Å^2^)

**Table:**

	*x*	*y*	*z*	*U*_iso_*/*U*_eq_	Occ. (<1)
Au	0.665037 (10)	0.182061 (8)	0.594476 (7)	0.02328 (4)	
P	0.82288 (7)	0.11891 (5)	0.69465 (5)	0.02176 (13)	
N1	−0.7853 (8)	0.3049 (6)	0.0355 (8)	0.0249 (19)	0.481 (4)
O1	−0.6757 (6)	0.2555 (6)	0.0027 (5)	0.0508 (16)	0.481 (4)
O2	−0.8285 (6)	0.3795 (4)	−0.0104 (4)	0.0542 (16)	0.481 (4)
O3	−0.8277 (5)	0.2750 (4)	0.1169 (4)	0.0433 (13)	0.481 (4)
N5	−0.1929 (19)	0.3572 (7)	0.1579 (14)	0.0312 (18)	0.481 (4)
H5	−0.2154	0.3451	0.2242	0.037*	0.481 (4)
C1	−0.3183 (7)	0.4390 (6)	0.0986 (5)	0.0272 (14)	0.481 (4)
H1A	−0.3322	0.5034	0.1316	0.033*	0.481 (4)
H1B	−0.2942	0.4552	0.0286	0.033*	0.481 (4)
C2	−0.4399 (6)	0.3793 (6)	0.1079 (5)	0.0415 (17)	0.481 (4)
H2A	−0.5135	0.4207	0.0791	0.062*	0.481 (4)
H2B	−0.4599	0.3615	0.1776	0.062*	0.481 (4)
H2C	−0.4251	0.3169	0.0732	0.062*	0.481 (4)
C3	−0.1635 (9)	0.2834 (7)	0.1254 (7)	0.0293 (18)	0.481 (4)
H3A	−0.2504	0.2552	0.1216	0.035*	0.481 (4)
H3B	−0.1239	0.2453	0.1834	0.035*	0.481 (4)
C4	−0.0910 (9)	0.2302 (8)	0.0440 (7)	0.058 (2)	0.481 (4)
H4A	−0.0945	0.1564	0.0570	0.087*	0.481 (4)
H4B	0.0004	0.2484	0.0409	0.087*	0.481 (4)
H4C	−0.1318	0.2512	−0.0189	0.087*	0.481 (4)
N1'	0.3290 (7)	0.3177 (6)	0.0098 (5)	0.0373 (13)	0.519 (4)
O1'	0.3781 (6)	0.2933 (5)	−0.0668 (5)	0.0708 (18)	0.519 (4)
O2'	0.3890 (6)	0.3670 (4)	0.0672 (3)	0.0610 (16)	0.519 (4)
O3'	0.2288 (17)	0.3057 (12)	0.0391 (12)	0.113 (5)	0.519 (4)
N5'	−0.2021 (18)	0.3913 (6)	0.1539 (13)	0.0312 (18)	0.519 (4)
H5'	−0.2375	0.3987	0.2168	0.037*	0.519 (4)
C1'	−0.3049 (9)	0.3885 (8)	0.0918 (8)	0.064 (3)	0.519 (4)
H1'A	−0.3527	0.3256	0.1036	0.076*	0.519 (4)
H1'B	−0.2777	0.3981	0.0216	0.076*	0.519 (4)
C2'	−0.3838 (8)	0.4842 (7)	0.1296 (7)	0.069 (2)	0.519 (4)
H2'A	−0.4691	0.4922	0.0987	0.104*	0.519 (4)
H2'B	−0.3350	0.5450	0.1126	0.104*	0.519 (4)
H2'C	−0.3965	0.4753	0.2012	0.104*	0.519 (4)
C3'	−0.1431 (14)	0.2479 (8)	0.1467 (8)	0.059 (3)	0.519 (4)
H3'A	−0.0571	0.2290	0.1760	0.071*	0.519 (4)
H3'B	−0.2092	0.1990	0.1694	0.071*	0.519 (4)
C4'	−0.1367 (9)	0.2780 (6)	0.0156 (6)	0.0492 (19)	0.519 (4)
H4'A	−0.1384	0.2152	−0.0185	0.074*	0.519 (4)
H4'B	−0.0560	0.3117	−0.0029	0.074*	0.519 (4)
H4'C	−0.2124	0.3236	−0.0032	0.074*	0.519 (4)
N2	−0.2745 (2)	0.40668 (18)	0.39973 (18)	0.0290 (5)	
O4	−0.2598 (2)	0.32863 (15)	0.35104 (16)	0.0399 (5)	
O5	−0.2412 (2)	0.40230 (17)	0.48672 (16)	0.0449 (6)	
O6	−0.3220 (2)	0.48678 (17)	0.35888 (18)	0.0484 (6)	
N3	0.5210 (2)	0.23408 (16)	0.49707 (16)	0.0218 (4)	
N4	0.2893 (3)	0.38262 (19)	0.27767 (19)	0.0354 (6)	
H4	0.293 (3)	0.362 (2)	0.2210 (16)	0.042*	
C5	−0.0876 (3)	0.4451 (3)	0.1452 (2)	0.0432 (8)	
H5A	−0.0406	0.4419	0.0808	0.052*	
H5B	−0.1337	0.5131	0.1473	0.052*	
C6	0.0088 (3)	0.4291 (3)	0.2274 (2)	0.0407 (8)	
H6A	0.0440	0.3574	0.2321	0.049*	
H6B	−0.0361	0.4433	0.2908	0.049*	
C7	0.1203 (3)	0.4995 (2)	0.2076 (2)	0.0292 (6)	
H7A	0.1600	0.4880	0.1418	0.035*	
H7B	0.0840	0.5709	0.2059	0.035*	
C8	0.2266 (3)	0.4855 (2)	0.2828 (2)	0.0288 (6)	
H8A	0.1857	0.4916	0.3499	0.035*	
C9	0.3271 (3)	0.5659 (2)	0.2646 (3)	0.0411 (8)	
H9A	0.3988	0.5513	0.3103	0.062*	
H9B	0.2854	0.6333	0.2749	0.062*	
H9C	0.3618	0.5649	0.1968	0.062*	
C10	0.3611 (2)	0.3343 (2)	0.3491 (2)	0.0252 (5)	
C11	0.3767 (2)	0.3752 (2)	0.43949 (19)	0.0239 (5)	
H11A	0.3325	0.4381	0.4536	0.029*	
C12	0.4565 (3)	0.3237 (2)	0.50772 (19)	0.0244 (5)	
H12A	0.4666	0.3545	0.5677	0.029*	
C13	0.5037 (2)	0.18783 (19)	0.41085 (18)	0.0206 (5)	
C14	0.5651 (2)	0.09074 (19)	0.39948 (19)	0.0235 (5)	
H14A	0.6166	0.0578	0.4505	0.028*	
C15	0.5501 (2)	0.04404 (19)	0.3141 (2)	0.0243 (5)	
Cl	0.63057 (7)	−0.07417 (5)	0.29880 (5)	0.03084 (15)	
C16	0.4726 (3)	0.0892 (2)	0.2383 (2)	0.0286 (6)	
H16A	0.4626	0.0554	0.1800	0.034*	
C17	0.4113 (3)	0.1831 (2)	0.2497 (2)	0.0294 (6)	
H17A	0.3580	0.2138	0.1987	0.035*	
C18	0.4253 (2)	0.2355 (2)	0.33543 (19)	0.0241 (5)	
C19	0.9009 (3)	0.00417 (19)	0.64813 (19)	0.0238 (5)	
C20	0.8238 (3)	−0.0623 (2)	0.6048 (2)	0.0294 (6)	
H20A	0.7337	−0.0443	0.5955	0.035*	
C21	0.8771 (3)	−0.1545 (2)	0.5750 (2)	0.0354 (7)	
H21A	0.8240	−0.1989	0.5444	0.042*	
C22	1.0076 (3)	−0.1817 (2)	0.5899 (2)	0.0356 (7)	
H22A	1.0434	−0.2461	0.5720	0.043*	
C23	1.0859 (3)	−0.1158 (2)	0.6308 (3)	0.0428 (8)	
H23A	1.1761	−0.1342	0.6397	0.051*	
C24	1.0332 (3)	−0.0222 (2)	0.6590 (2)	0.0352 (7)	
H24A	1.0879	0.0238	0.6858	0.042*	
C25	0.9531 (3)	0.20251 (19)	0.71033 (19)	0.0224 (5)	
C26	0.9777 (3)	0.2794 (2)	0.6378 (2)	0.0284 (6)	
H26A	0.9194	0.2936	0.5851	0.034*	
C27	1.0875 (3)	0.3353 (2)	0.6425 (2)	0.0363 (7)	
H27A	1.1058	0.3862	0.5919	0.044*	
C28	1.1707 (3)	0.3170 (2)	0.7209 (2)	0.0380 (7)	
H28A	1.2464	0.3548	0.7233	0.046*	
C29	1.1439 (3)	0.2440 (2)	0.7953 (2)	0.0348 (7)	
H29A	1.1995	0.2330	0.8499	0.042*	
C30	1.0360 (3)	0.1870 (2)	0.7902 (2)	0.0295 (6)	
H30A	1.0179	0.1367	0.8414	0.035*	
C31	0.7642 (2)	0.0799 (2)	0.81795 (19)	0.0246 (5)	
C32	0.6619 (3)	0.1374 (2)	0.8616 (2)	0.0294 (6)	
H32A	0.6227	0.1955	0.8263	0.035*	
C33	0.6179 (3)	0.1098 (3)	0.9562 (2)	0.0417 (8)	
H33A	0.5484	0.1490	0.9858	0.050*	
C34	0.6748 (3)	0.0252 (3)	1.0080 (2)	0.0452 (8)	
H34A	0.6456	0.0073	1.0735	0.054*	
C35	0.7737 (3)	−0.0329 (3)	0.9646 (2)	0.0466 (9)	
H35A	0.8114	−0.0916	0.9999	0.056*	
C36	0.8185 (3)	−0.0065 (2)	0.8699 (2)	0.0350 (7)	
H36A	0.8864	−0.0472	0.8403	0.042*	

### Atomic displacement parameters (Å^2^)

**Table:**

	*U*^11^	*U*^22^	*U*^33^	*U*^12^	*U*^13^	*U*^23^
Au	0.02601 (6)	0.02376 (5)	0.02042 (5)	−0.00079 (4)	−0.00528 (4)	−0.00233 (4)
P	0.0256 (3)	0.0196 (3)	0.0201 (3)	0.0005 (3)	−0.0027 (3)	−0.0022 (2)
N1	0.017 (3)	0.023 (4)	0.038 (5)	−0.010 (3)	0.004 (3)	−0.020 (3)
O1	0.039 (3)	0.067 (5)	0.049 (4)	0.010 (3)	−0.014 (3)	−0.022 (3)
O2	0.075 (4)	0.049 (3)	0.035 (3)	0.023 (3)	−0.007 (3)	0.004 (2)
O3	0.053 (3)	0.047 (3)	0.029 (3)	0.011 (2)	−0.013 (2)	−0.004 (2)
N5	0.036 (3)	0.033 (6)	0.0265 (19)	−0.015 (5)	−0.0003 (17)	−0.006 (5)
C1	0.026 (3)	0.035 (4)	0.021 (3)	0.004 (3)	−0.003 (2)	−0.005 (3)
C2	0.028 (3)	0.052 (4)	0.046 (4)	−0.013 (3)	−0.008 (3)	−0.005 (3)
C3	0.035 (4)	0.027 (5)	0.027 (4)	−0.016 (3)	0.002 (3)	−0.001 (3)
C4	0.052 (6)	0.066 (7)	0.057 (6)	−0.005 (5)	0.003 (4)	−0.012 (5)
N1'	0.041 (4)	0.040 (3)	0.030 (3)	−0.001 (3)	−0.009 (3)	0.000 (3)
O1'	0.065 (4)	0.102 (5)	0.047 (3)	0.013 (3)	−0.012 (3)	−0.031 (3)
O2'	0.085 (4)	0.068 (4)	0.033 (3)	−0.022 (3)	−0.013 (3)	−0.004 (2)
O3'	0.128 (12)	0.129 (11)	0.080 (10)	0.012 (9)	−0.019 (8)	0.005 (8)
N5'	0.036 (3)	0.033 (6)	0.0265 (19)	−0.015 (5)	−0.0003 (17)	−0.006 (5)
C1'	0.046 (5)	0.065 (6)	0.084 (7)	0.005 (5)	−0.008 (4)	−0.035 (6)
C2'	0.048 (5)	0.095 (7)	0.064 (6)	0.005 (5)	−0.013 (4)	−0.004 (5)
C3'	0.107 (8)	0.036 (6)	0.034 (6)	−0.009 (5)	0.008 (5)	−0.003 (4)
C4'	0.063 (5)	0.045 (5)	0.041 (4)	−0.003 (4)	0.006 (4)	−0.019 (4)
N2	0.0287 (12)	0.0258 (12)	0.0328 (13)	−0.0032 (10)	0.0054 (10)	−0.0056 (10)
O4	0.0540 (14)	0.0294 (11)	0.0370 (12)	−0.0050 (10)	0.0130 (10)	−0.0117 (9)
O5	0.0638 (15)	0.0386 (12)	0.0328 (12)	0.0051 (11)	−0.0117 (11)	−0.0076 (10)
O6	0.0619 (16)	0.0328 (12)	0.0491 (15)	0.0070 (11)	−0.0092 (12)	0.0009 (11)
N3	0.0228 (11)	0.0217 (11)	0.0211 (11)	−0.0007 (8)	−0.0012 (8)	−0.0028 (8)
N4	0.0446 (15)	0.0291 (13)	0.0328 (14)	0.0132 (11)	−0.0171 (12)	−0.0108 (11)
C5	0.0322 (16)	0.067 (2)	0.0310 (17)	−0.0116 (15)	−0.0027 (13)	0.0044 (15)
C6	0.0334 (16)	0.059 (2)	0.0311 (16)	−0.0192 (15)	−0.0072 (13)	0.0057 (15)
C7	0.0296 (14)	0.0244 (14)	0.0326 (15)	0.0005 (11)	−0.0038 (12)	0.0040 (11)
C8	0.0270 (14)	0.0220 (13)	0.0377 (16)	0.0044 (11)	−0.0064 (12)	−0.0065 (11)
C9	0.0358 (17)	0.0388 (18)	0.051 (2)	−0.0078 (14)	−0.0045 (15)	−0.0112 (15)
C10	0.0225 (13)	0.0260 (13)	0.0276 (14)	0.0022 (10)	−0.0064 (11)	−0.0060 (11)
C11	0.0236 (13)	0.0218 (12)	0.0271 (14)	0.0013 (10)	−0.0017 (10)	−0.0079 (10)
C12	0.0272 (13)	0.0250 (13)	0.0220 (13)	−0.0041 (10)	−0.0009 (10)	−0.0056 (10)
C13	0.0198 (12)	0.0216 (12)	0.0204 (12)	−0.0013 (9)	0.0002 (9)	−0.0016 (10)
C14	0.0217 (12)	0.0242 (13)	0.0241 (13)	0.0018 (10)	−0.0008 (10)	−0.0013 (10)
C15	0.0213 (12)	0.0227 (13)	0.0291 (14)	0.0019 (10)	0.0013 (10)	−0.0061 (11)
Cl	0.0286 (3)	0.0248 (3)	0.0391 (4)	0.0053 (3)	0.0004 (3)	−0.0085 (3)
C16	0.0318 (14)	0.0289 (14)	0.0264 (14)	0.0016 (11)	−0.0039 (11)	−0.0110 (11)
C17	0.0323 (14)	0.0305 (15)	0.0256 (14)	0.0058 (12)	−0.0100 (11)	−0.0063 (11)
C18	0.0236 (13)	0.0246 (13)	0.0247 (13)	0.0019 (10)	−0.0053 (10)	−0.0066 (10)
C19	0.0305 (14)	0.0215 (12)	0.0190 (12)	−0.0017 (10)	0.0046 (10)	−0.0005 (10)
C20	0.0379 (16)	0.0239 (13)	0.0263 (14)	−0.0007 (12)	−0.0026 (12)	−0.0020 (11)
C21	0.0530 (19)	0.0236 (14)	0.0304 (16)	−0.0055 (13)	0.0011 (14)	−0.0058 (12)
C22	0.0485 (18)	0.0225 (14)	0.0348 (17)	−0.0008 (13)	0.0175 (14)	−0.0048 (12)
C23	0.0317 (16)	0.0336 (17)	0.062 (2)	−0.0004 (13)	0.0200 (15)	−0.0089 (15)
C24	0.0295 (15)	0.0284 (15)	0.0488 (19)	−0.0063 (12)	0.0112 (13)	−0.0113 (13)
C25	0.0253 (13)	0.0210 (12)	0.0213 (13)	0.0001 (10)	−0.0001 (10)	−0.0046 (10)
C26	0.0330 (15)	0.0281 (14)	0.0243 (14)	−0.0010 (11)	−0.0017 (11)	−0.0039 (11)
C27	0.0391 (17)	0.0326 (16)	0.0377 (17)	−0.0075 (13)	0.0088 (14)	−0.0051 (13)
C28	0.0296 (15)	0.0389 (17)	0.0482 (19)	−0.0058 (13)	0.0041 (14)	−0.0204 (15)
C29	0.0290 (15)	0.0386 (17)	0.0389 (17)	0.0035 (13)	−0.0087 (13)	−0.0187 (14)
C30	0.0359 (15)	0.0286 (14)	0.0243 (14)	0.0030 (12)	−0.0037 (12)	−0.0069 (11)
C31	0.0235 (13)	0.0289 (14)	0.0219 (13)	−0.0018 (10)	−0.0021 (10)	−0.0048 (11)
C32	0.0295 (14)	0.0279 (14)	0.0315 (15)	−0.0012 (11)	−0.0007 (12)	−0.0068 (12)
C33	0.0378 (17)	0.0485 (19)	0.0396 (18)	−0.0014 (15)	0.0138 (14)	−0.0165 (15)
C34	0.0420 (19)	0.067 (2)	0.0250 (16)	−0.0028 (17)	0.0085 (14)	0.0003 (15)
C35	0.0392 (18)	0.061 (2)	0.0348 (18)	0.0052 (16)	0.0036 (14)	0.0191 (16)
C36	0.0309 (15)	0.0408 (17)	0.0303 (16)	0.0082 (13)	0.0044 (12)	0.0061 (13)

### Geometric parameters (Å, °)

**Table:**

Au—N3	2.070 (2)	C7—C8	1.513 (4)
Au—P	2.2338 (7)	C7—H7A	0.9900
P—C25	1.809 (3)	C7—H7B	0.9900
P—C19	1.814 (3)	C8—C9	1.526 (4)
P—C31	1.816 (3)	C8—H8A	1.0000
N1—O2	1.197 (12)	C9—H9A	0.9800
N1—O3	1.226 (12)	C9—H9B	0.9800
N1—O1	1.343 (11)	C9—H9C	0.9800
N5—C3	1.119 (17)	C10—C11	1.398 (4)
N5—C5	1.628 (16)	C10—C18	1.445 (3)
N5—C1	1.801 (18)	C11—C12	1.373 (4)
N5—H5	0.9300	C11—H11A	0.9500
C1—C2	1.511 (10)	C12—H12A	0.9500
C1—H1A	0.9900	C13—C14	1.409 (3)
C1—H1B	0.9900	C13—C18	1.413 (3)
C2—H2A	0.9800	C14—C15	1.373 (4)
C2—H2B	0.9800	C14—H14A	0.9500
C2—H2C	0.9800	C15—C16	1.400 (4)
C3—C4	1.507 (14)	C15—Cl	1.742 (3)
C3—H3A	0.9900	C16—C17	1.371 (4)
C3—H3B	0.9900	C16—H16A	0.9500
C4—H4A	0.9800	C17—C18	1.415 (4)
C4—H4B	0.9800	C17—H17A	0.9500
C4—H4C	0.9800	C19—C24	1.388 (4)
N1'—O3'	1.105 (16)	C19—C20	1.389 (4)
N1'—O1'	1.201 (9)	C20—C21	1.385 (4)
N1'—O2'	1.251 (8)	C20—H20A	0.9500
N5'—C1'	1.38 (2)	C21—C22	1.381 (4)
N5'—C5	1.405 (17)	C21—H21A	0.9500
N5'—C3'	1.959 (14)	C22—C23	1.377 (4)
N5'—H5'	0.9300	C22—H22A	0.9500
C1'—C2'	1.567 (12)	C23—C24	1.392 (4)
C1'—H1'A	0.9900	C23—H23A	0.9500
C1'—H1'B	0.9900	C24—H24A	0.9500
C2'—H2'A	0.9800	C25—C26	1.393 (4)
C2'—H2'B	0.9800	C25—C30	1.398 (4)
C2'—H2'C	0.9800	C26—C27	1.388 (4)
C3'—C4'	1.803 (14)	C26—H26A	0.9500
C3'—H3'A	0.9900	C27—C28	1.386 (5)
C3'—H3'B	0.9900	C27—H27A	0.9500
C4'—H4'A	0.9800	C28—C29	1.380 (5)
C4'—H4'B	0.9800	C28—H28A	0.9500
C4'—H4'C	0.9800	C29—C30	1.380 (4)
N2—O6	1.238 (3)	C29—H29A	0.9500
N2—O5	1.241 (3)	C30—H30A	0.9500
N2—O4	1.264 (3)	C31—C36	1.393 (4)
N3—C12	1.334 (3)	C31—C32	1.399 (4)
N3—C13	1.384 (3)	C32—C33	1.384 (4)
N4—C10	1.340 (3)	C32—H32A	0.9500
N4—C8	1.471 (3)	C33—C34	1.385 (5)
N4—H4	0.839 (18)	C33—H33A	0.9500
C5—C6	1.513 (4)	C34—C35	1.377 (5)
C5—H5A	0.9900	C34—H34A	0.9500
C5—H5B	0.9900	C35—C36	1.383 (4)
C6—C7	1.521 (4)	C35—H35A	0.9500
C6—H6A	0.9900	C36—H36A	0.9500
C6—H6B	0.9900		
			
N3—Au—P	176.94 (6)	N4—C8—C9	110.7 (2)
C25—P—C19	105.97 (12)	C7—C8—C9	111.3 (2)
C25—P—C31	105.86 (12)	N4—C8—H8A	108.8
C19—P—C31	104.78 (12)	C7—C8—H8A	108.8
C25—P—Au	115.47 (9)	C9—C8—H8A	108.8
C19—P—Au	110.06 (9)	C8—C9—H9A	109.5
C31—P—Au	113.88 (9)	C8—C9—H9B	109.5
O2—N1—O3	123.8 (7)	H9A—C9—H9B	109.5
O2—N1—O1	119.5 (9)	C8—C9—H9C	109.5
O3—N1—O1	116.3 (9)	H9A—C9—H9C	109.5
C3—N5—C5	116.5 (15)	H9B—C9—H9C	109.5
C3—N5—C1	118.2 (15)	N4—C10—C11	122.5 (2)
C5—N5—C1	92.3 (6)	N4—C10—C18	120.5 (2)
C3—N5—H5	109.6	C11—C10—C18	116.9 (2)
C5—N5—H5	109.6	C12—C11—C10	119.5 (2)
C1—N5—H5	109.6	C12—C11—H11A	120.3
C2—C1—N5	105.1 (7)	C10—C11—H11A	120.3
C2—C1—H1A	110.7	N3—C12—C11	125.6 (2)
N5—C1—H1A	110.7	N3—C12—H12A	117.2
C2—C1—H1B	110.7	C11—C12—H12A	117.2
N5—C1—H1B	110.7	N3—C13—C14	118.6 (2)
H1A—C1—H1B	108.8	N3—C13—C18	121.4 (2)
N5—C3—C4	147.4 (12)	C14—C13—C18	120.0 (2)
N5—C3—H3A	99.9	C15—C14—C13	119.5 (2)
C4—C3—H3A	99.9	C15—C14—H14A	120.3
N5—C3—H3B	99.9	C13—C14—H14A	120.3
C4—C3—H3B	99.9	C14—C15—C16	121.8 (2)
H3A—C3—H3B	104.2	C14—C15—Cl	119.3 (2)
O3'—N1'—O1'	128.3 (11)	C16—C15—Cl	118.9 (2)
O3'—N1'—O2'	110.2 (11)	C17—C16—C15	118.8 (2)
O1'—N1'—O2'	121.5 (7)	C17—C16—H16A	120.6
C1'—N5'—C5	131.5 (12)	C15—C16—H16A	120.6
C1'—N5'—C3'	95.6 (9)	C16—C17—C18	121.8 (3)
C5—N5'—C3'	105.3 (10)	C16—C17—H17A	119.1
C1'—N5'—H5'	107.3	C18—C17—H17A	119.1
C5—N5'—H5'	107.3	C13—C18—C17	118.1 (2)
C3'—N5'—H5'	107.3	C13—C18—C10	119.2 (2)
N5'—C1'—C2'	95.8 (8)	C17—C18—C10	122.6 (2)
N5'—C1'—H1'A	112.6	C24—C19—C20	118.9 (2)
C2'—C1'—H1'A	112.6	C24—C19—P	122.4 (2)
N5'—C1'—H1'B	112.6	C20—C19—P	118.6 (2)
C2'—C1'—H1'B	112.6	C21—C20—C19	120.7 (3)
H1'A—C1'—H1'B	110.1	C21—C20—H20A	119.7
C1'—C2'—H2'A	109.5	C19—C20—H20A	119.7
C1'—C2'—H2'B	109.5	C22—C21—C20	119.8 (3)
H2'A—C2'—H2'B	109.5	C22—C21—H21A	120.1
C1'—C2'—H2'C	109.5	C20—C21—H21A	120.1
H2'A—C2'—H2'C	109.5	C23—C22—C21	120.2 (3)
H2'B—C2'—H2'C	109.5	C23—C22—H22A	119.9
C4'—C3'—N5'	84.7 (7)	C21—C22—H22A	119.9
C4'—C3'—H3'A	114.5	C22—C23—C24	120.0 (3)
N5'—C3'—H3'A	114.5	C22—C23—H23A	120.0
C4'—C3'—H3'B	114.5	C24—C23—H23A	120.0
N5'—C3'—H3'B	114.5	C19—C24—C23	120.3 (3)
H3'A—C3'—H3'B	111.6	C19—C24—H24A	119.9
C3'—C4'—H4'A	109.5	C23—C24—H24A	119.9
C3'—C4'—H4'B	109.5	C26—C25—C30	119.0 (2)
H4'A—C4'—H4'B	109.5	C26—C25—P	119.4 (2)
C3'—C4'—H4'C	109.5	C30—C25—P	121.4 (2)
H4'A—C4'—H4'C	109.5	C27—C26—C25	119.9 (3)
H4'B—C4'—H4'C	109.5	C27—C26—H26A	120.1
O6—N2—O5	121.2 (2)	C25—C26—H26A	120.1
O6—N2—O4	118.8 (3)	C28—C27—C26	120.3 (3)
O5—N2—O4	120.0 (2)	C28—C27—H27A	119.9
C12—N3—C13	117.2 (2)	C26—C27—H27A	119.9
C12—N3—Au	119.89 (17)	C29—C28—C27	120.2 (3)
C13—N3—Au	121.94 (16)	C29—C28—H28A	119.9
C10—N4—C8	124.3 (2)	C27—C28—H28A	119.9
C10—N4—H4	120 (2)	C30—C29—C28	119.8 (3)
C8—N4—H4	114 (2)	C30—C29—H29A	120.1
N5'—C5—C6	118.1 (7)	C28—C29—H29A	120.1
C6—C5—N5	109.3 (7)	C29—C30—C25	120.7 (3)
N5'—C5—H5A	114.5	C29—C30—H30A	119.7
C6—C5—H5A	109.8	C25—C30—H30A	119.7
N5—C5—H5A	109.8	C36—C31—C32	119.2 (3)
N5'—C5—H5B	95.0	C36—C31—P	121.4 (2)
C6—C5—H5B	109.8	C32—C31—P	119.4 (2)
N5—C5—H5B	109.8	C33—C32—C31	120.0 (3)
H5A—C5—H5B	108.3	C33—C32—H32A	120.0
C5—C6—C7	109.9 (3)	C31—C32—H32A	120.0
C5—C6—H6A	109.7	C32—C33—C34	120.2 (3)
C7—C6—H6A	109.7	C32—C33—H33A	119.9
C5—C6—H6B	109.7	C34—C33—H33A	119.9
C7—C6—H6B	109.7	C35—C34—C33	120.0 (3)
H6A—C6—H6B	108.2	C35—C34—H34A	120.0
C8—C7—C6	114.3 (2)	C33—C34—H34A	120.0
C8—C7—H7A	108.7	C34—C35—C36	120.5 (3)
C6—C7—H7A	108.7	C34—C35—H35A	119.8
C8—C7—H7B	108.7	C36—C35—H35A	119.8
C6—C7—H7B	108.7	C35—C36—C31	120.0 (3)
H7A—C7—H7B	107.6	C35—C36—H36A	120.0
N4—C8—C7	108.4 (2)	C31—C36—H36A	120.0
			
C3—N5—C1—C2	63.6 (16)	N4—C10—C18—C13	178.0 (3)
C5—N5—C1—C2	−174.5 (7)	C11—C10—C18—C13	−2.7 (4)
C5—N5—C3—C4	−34 (3)	N4—C10—C18—C17	−3.0 (4)
C1—N5—C3—C4	74 (3)	C11—C10—C18—C17	176.3 (3)
C5—N5'—C1'—C2'	−85.5 (15)	C25—P—C19—C24	20.4 (3)
C3'—N5'—C1'—C2'	158.3 (8)	C31—P—C19—C24	−91.3 (2)
C1'—N5'—C3'—C4'	54.7 (10)	Au—P—C19—C24	145.9 (2)
C5—N5'—C3'—C4'	−81.1 (10)	C25—P—C19—C20	−163.2 (2)
C1'—N5'—C5—C6	−178.7 (11)	C31—P—C19—C20	85.1 (2)
C3'—N5'—C5—C6	−66.6 (11)	Au—P—C19—C20	−37.7 (2)
C1'—N5'—C5—N5	−122 (6)	C24—C19—C20—C21	1.5 (4)
C3'—N5'—C5—N5	−10 (5)	P—C19—C20—C21	−175.1 (2)
C3—N5—C5—N5'	146 (7)	C19—C20—C21—C22	1.2 (4)
C1—N5—C5—N5'	23 (5)	C20—C21—C22—C23	−2.6 (5)
C3—N5—C5—C6	−85.2 (15)	C21—C22—C23—C24	1.3 (5)
C1—N5—C5—C6	151.6 (6)	C20—C19—C24—C23	−2.8 (4)
N5'—C5—C6—C7	−175.9 (7)	P—C19—C24—C23	173.6 (2)
N5—C5—C6—C7	170.8 (7)	C22—C23—C24—C19	1.4 (5)
C5—C6—C7—C8	−176.8 (3)	C19—P—C25—C26	98.4 (2)
C10—N4—C8—C7	−161.2 (3)	C31—P—C25—C26	−150.7 (2)
C10—N4—C8—C9	76.5 (4)	Au—P—C25—C26	−23.7 (2)
C6—C7—C8—N4	64.2 (3)	C19—P—C25—C30	−76.8 (2)
C6—C7—C8—C9	−173.9 (3)	C31—P—C25—C30	34.1 (2)
C8—N4—C10—C11	4.3 (5)	Au—P—C25—C30	161.09 (19)
C8—N4—C10—C18	−176.5 (3)	C30—C25—C26—C27	3.7 (4)
N4—C10—C11—C12	−177.1 (3)	P—C25—C26—C27	−171.6 (2)
C18—C10—C11—C12	3.6 (4)	C25—C26—C27—C28	−2.0 (4)
C13—N3—C12—C11	−1.6 (4)	C26—C27—C28—C29	−0.9 (5)
Au—N3—C12—C11	167.6 (2)	C27—C28—C29—C30	1.9 (4)
C10—C11—C12—N3	−1.6 (4)	C28—C29—C30—C25	−0.2 (4)
C12—N3—C13—C14	−176.8 (2)	C26—C25—C30—C29	−2.7 (4)
Au—N3—C13—C14	14.3 (3)	P—C25—C30—C29	172.5 (2)
C12—N3—C13—C18	2.6 (4)	C25—P—C31—C36	−89.6 (3)
Au—N3—C13—C18	−166.36 (19)	C19—P—C31—C36	22.2 (3)
N3—C13—C14—C15	−179.4 (2)	Au—P—C31—C36	142.5 (2)
C18—C13—C14—C15	1.2 (4)	C25—P—C31—C32	90.7 (2)
C13—C14—C15—C16	−1.5 (4)	C19—P—C31—C32	−157.5 (2)
C13—C14—C15—Cl	177.96 (19)	Au—P—C31—C32	−37.2 (2)
C14—C15—C16—C17	0.7 (4)	C36—C31—C32—C33	1.5 (4)
Cl—C15—C16—C17	−178.8 (2)	P—C31—C32—C33	−178.7 (2)
C15—C16—C17—C18	0.5 (4)	C31—C32—C33—C34	0.0 (5)
N3—C13—C18—C17	−179.5 (2)	C32—C33—C34—C35	−1.3 (5)
C14—C13—C18—C17	−0.1 (4)	C33—C34—C35—C36	1.1 (6)
N3—C13—C18—C10	−0.4 (4)	C34—C35—C36—C31	0.4 (5)
C14—C13—C18—C10	178.9 (2)	C32—C31—C36—C35	−1.7 (5)
C16—C17—C18—C13	−0.8 (4)	P—C31—C36—C35	178.5 (3)
C16—C17—C18—C10	−179.8 (3)		

### Hydrogen-bond geometry (Å, °)

**Table:**

*D*—H···*A*	*D*—H	H···*A*	*D*···*A*	*D*—H···*A*
N5—H5···O4	0.93	1.77	2.70 (2)	177
N5'—H5'···O4	0.93	2.00	2.81 (2)	144
N4—H4···O2'	0.84 (2)	2.28 (2)	3.030 (6)	149 (3)
N4—H4···O3^i^	0.84 (2)	2.32 (3)	3.013 (5)	140 (3)

Symmetry codes: (i) *x*+1, *y*, *z*.
